# Predictive diagnosis of endometrial hyperplasia and personalized therapeutic strategy in women of fertile age

**DOI:** 10.1186/1878-5085-4-24

**Published:** 2013-12-06

**Authors:** Vadym M Goncharenko, Vasyl A Beniuk, Olga V Kalenska, Olga M Demchenko, Mykola Ya Spivak, Rostyslav V Bubnov

**Affiliations:** 1 Clinical Hospital ‘Pheophania’ of State Affairs Department, Zabolotny str., 21, Kyiv 03680, Ukraine; 2 Third Department of Obstetrics and Gynecology, Bogomolets National Medical University, Kyiv 01601, Ukraine; 3 JSC SPC ‘DiaprofMed’, Svitlycky str., 35, Kyiv 04123, Ukraine; 4 Zabolotny Institute of Microbiology and Virology, National Academy of Sciences of Ukraine, Zabolotny Str., 154, Kyiv 03680, Ukraine; 5 Center of Ultrasound Diagnostics and Interventional Sonography, Clinical Hospital ‘Pheophania’ of State Affairs Department, Zabolotny str., 21, Kyiv 03680, Ukraine

**Keywords:** Predictive, Preventive, Personalized medicine, Women’s health, Endometrial hyperplasia, Endometrial receptors, Transvaginal ultrasonography, Sonoelastography, Fertile age women, Endoscopic treatment, Hysteroresection

## Abstract

**Introduction:**

Endometrial hyperplasia has a high risk for malignant transformation and relapses; existing mini-invasive treatments may lead to irrevocable endometrium destruction. The *aims* were to analyze receptor systems in endometrial hyperplasia, to evaluate the capabilities of ultrasonography, sonoelastography for diagnosis and treatment control, and to develop treatment algorithm.

**Materials and methods:**

We included 313 women (20–45 years), assessed into the following: group 1 (*n* = 112) with glandular cystic hyperplasia, group 2 (*n* = 98) endometrial polyps, and group 3 (*n* = 103) atypical hyperplasia; and 82 controls who have undergone hysteroscopy before *in vitro* fertilization in tubal origin infertility were also included. Patients underwent clinical examination, transvaginal ultrasound, immunohistochemical study, and hormonal therapy/hysteroresectoscopy.

**Results:**

In patients with glandular hyperplasia, we registered increase of endometrium estrogen receptors (75.6% in the epithelium and 30.9% in the stroma; in controls, 43.3% and 29.6%, respectively); in polyps, there was a significant estrogen receptor increase in the stroma (48.2% vs 29.6% in controls), and in atypical hyperplasia, progesterone receptors significantly increased in the stroma. Ki-67 increased (40% to 50%) in the epithelium without changes in the stroma. Ultrasound has a sensitivity of 96% and a specificity of 85% for early detection of endometrial pathology and prediction outcome of intervention, and sonoelastography has a sensitivity of 91% and a specificity of 83% for polyp diagnosis. Personalized treatment was effective in 88.8%, relapse was diagnosed in 11.2% after 6 months, and conservative treatment of atypical hyperplasia was effective in 45%: in 25.8%, ablative hysteroresectoscopy was performed, while in 22.6% with comorbidities, hystero/oophorectomies were performed.

**Conclusions:**

The evaluation of receptor status with ultrasound data in patients with endometrial hyperplasia allows for a clear definition of the treatment policy, avoidance of relapse, treatment optimization, and observation of such patients.

## Overview

### Current evidence for predictive, preventive, and personalized strategy for endometrial hyperplasia

Women's health and gender-related pathology remain the priorities for predictive, preventive, and personalized medicine (PPPM) [1,2]. Relevance of endometrial hyperplasia (EH) study is primarily due to a high risk for malignant transformation and the problems associated with menstrual irregularities, dysfunctional uterine bleeding, and anemia in women. Endometrial hyperplasia has a significant place in the structure of gynecological morbidity in women of reproductive age and is one of the most frequent causes of hospitalization in gynecology hospital (10% to 18%) [3].

Endometrial hyperplasia may cause endometrial cancer in up to 50% of cases [4]. The incidence of endometrial adenocarcinoma, which ranks first among genital malignancies, not only has remained high but in recent years has tended to significantly increase in many countries, including Ukraine, and according to long-term prognosis, it will not diminish anytime soon [3,5]. Endometrial cancers are the most common gynecologic cancers in developed countries [6]; therefore, careful search for malignancy, particularly in women with multiple risk factors, is advised by many researchers in daily practice [6,7]. With the high rate of endometrial hyperplasia recurrence, the risks of malignancy require further improvement and new approaches to diagnosis and treat of this disease should be found [8]. Additional studies on histological features and immunohistochemical profiles are needed to find associations between endometrioid and high-grade endometrial carcinoma and endometrial pathology. Differences in the immunohistochemical expression of p53, B cell lymphoma 2 (Bcl-2), bax, estrogen receptor (ER), and progesterone receptor (PR), androgen receptor (AR), progesterone receptor antagonists (PA), etc. should be properly assessed to find *the most common diagnostic pitfalls and helpful morphological and immunohistochemical markers.*

### Endometrial hyperplasia in reproductive-aged women

The endometrium of reproductive-aged women undergoes cyclic developmental changes in response to the steroids - estrogen and progesterone.

The highest score of *ERs* and *PRs is observed in the epithelial and stromal cells* of the normal endometrial uterine at the early proliferative phase; then, throughout the secretory phase, the ER and PR scores decline. In typical endometriotic lesions, the ER and PR scores are constantly high, but they are independent of the menstrual cycle. The expression pattern of ER mRNA is reported mostly in parallel with that of ERs. In typical endometriosis, ERs and PRs are found in both glandular epithelial cells and in their surrounding stromal cells. Expression of ER mRNA was found in typical endometriotic peritonea and in the pelvic peritoneum with columnar epithelial cells, but not in the normal pelvic peritoneum (mesothelium). Estrogen receptors and PRs were found negative in the mesothelium but were positive in the nuclei of fibroblasts in the connective tissue [9].

Gregory et al. postulated that an increased in the coactivator expression may render the endometrium to be more sensitive to estrogen [10]. Specific coactivator expression patterns were found in the fertile endometrium and in anovulatory (proliferative) and clomiphene-induced ovulatory (secretory) women with polycystic ovarian syndrome (PCOS), who have a higher likelihood of developing estrogen-induced endometrial hyperplasia and cancer. Women with PCOS exhibited elevated levels of amplified in breast cancer-1 (AIB1) and transcriptional intermediary factor-2 expression in both the epithelial and stromal cells [10]. Their receptors are regulated by steroid receptor coactivators of the p160 family, namely steroid receptor coactivator-1, AIB1, and transcriptional intermediary factor-2, in the human endometrium obtained prospectively from normal fertile women throughout the menstrual cycle. Glandular AIB1 increases in the late secretory phase [10]. The authors described an increased expression of ERα (an estrogen-induced gene product) during the menstrual cycle in the PCOS endometrium and overexpression of p160 in the endometrium of women with PCOS. These data explain the poor reproductive performance observed in PCOS and the increased incidence of endometrial hyperplasia and cancer noted in this group of women.

The effects of estradiol (E), progesterone (P), and PA were studied on the endometrium of rhesus macaque [11]. Ovariectomized macaques were treated with implants of E and P to induce precisely controlled, artificial menstrual cycles. During these cycles, treatment with E alone induces an artificial endometrial epithelial cell proliferation and increased expression of stromal and epithelial ER and PR. Androgen receptor E in the endometrial stroma is also upregulated. Progesterone acts on the E-primed endometrium to induce secretory differentiation and causes suppression of epithelial and stromal ER, epithelial PR, and stromal AR in the functionalis zone. However, epithelial ER and PR are retained in the basalis zone during the secretory phase. When potent PA are administered acutely at the end of an E(2) + P-induced cycle, menses typically ensues similarly to P withdrawal at the end of the menstrual cycle. When PAs are administered chronically, there is significant blockage of all P-dependent effects including upregulation of ER, PR, and AR and suppression of glandular secretory function. However, chronic PA administration also inhibits estrogen-dependent endometrial cell proliferation and growth. This experimental data endometrial anti-proliferative effect is the basis of the clinical use of PA to control various diseases such as endometriosis [11].

### Atypical endometrial hyperplasia

Atypical endometrial hyperplasia (AEH) has the highest cancer threat; the prevalence of endometrial carcinoma in patients who had a community hospital biopsy diagnosis of AEH was high (42.6%). When considering management strategies for women who have a biopsy diagnosis of AEH, clinicians and patients should take into account the considerable rate of concurrent carcinoma [12]. Malignant tumors after AEH diagnosis demonstrate features of good prognosis with endometrioid morphology, lower grade, and early stage, although the overall positive predictive value of AEH is expected at 37% to 48% in the current routine practice [13].

#### Endometrial cancer

Endometrial cancer is frequently seen in women with post-menopausal bleeding and endometrial hypertrophy in ultrasound examination, especially when the endometrial image is non-homogenous and irregular. However, the rarest endometrial cancers were affirmed in post-menopausal women with the ultrasound image of fluid in the uterine cavity with thin endometrium [14].

Endometrial cancers are classified into *types I and II* based on light microscopic appearance, clinical behavior, and epidemiology. This classification of endometrial cancers considers genetic analysis, and histologic subtypes are underscored by systematic changes in a limited set of genes. Common genetic changes in endometrioid endometrial cancers include, but are not limited to, microsatellite instability or specific mutation of PTEN, K-ras,12,22-28, and β-catenin genes [15].

Type I comprises 70% to 80% of newly diagnosed cases of endometrial cancer, is associated with unopposed estrogen exposure, and is often preceded by a pre-malignant disease.

Type II of endometrial cancers has a non-endometrioid histology (usually papillary serous or clear cell) with an aggressive clinical course. Hormonal risk factors have not been identified, and there is no readily observed pre-malignant phase. Combined molecular, histomorphometric, and clinical outcome analysis of premalignant lesions has provided a clearer multidisciplinary definition of endometrial pre-cancers, known as EIN. Genetic and endocrine disease mechanisms have been integrated into a multistep model for oncogenesis in which hormonal exposures act as selection factors for mutated endometrial cells [15].

The findings of Kounelis et al. [16] indicate the differences in immunohistochemical profiles of endometrioid and serous carcinomas. Thus, uterine papillary serous adenocarcinomas (UPSA) show a significantly higher p53 expression than uterine endometrioid adenocarcinomas; there is no significant difference in Bcl-2 and bax expression between both histologic types. Overexpression of p53 is associated with high-grade endometrioid carcinoma and advanced-stage tumors, while ER and PR expressions were associated with low-grade and early-stage tumors. Bcl-2 immunopositivity is more common in low-grade, early-stage adenocarcinomas rather than in high-grade, advanced-stage adenocarcinomas, but the difference was not statistically significant. Bax immunopositivity is associated with well-differentiated and early-stage tumors. There was a significant inverse relationship between bax and p53 reactivity, especially in tumors of endometrioid type [16,17]. Early detection of p53 nuclear accumulation may help to identify precursor lesions of UPSA. Bcl-2 persistence is frequently associated with endometrial carcinoma, and failure to inactivate Bcl-2 expression probably is related to the development of endometrial carcinoma [17].

The presence of testosterone receptors in estrogen receptor-positive endometrial carcinomas may be involved in the mechanism of cell proliferation in these tumors. The strong staining reaction for testosterone receptors in the endometrial glands can be considered one of the features of invasive malignancy [18].

Bartoschet et al. [19] suggest differential diagnosis between the different subtypes of endometrial carcinomas including (1) endometrioid versus serous glandular carcinoma, (2) papillary endometrioid (not otherwise specified, villoglandular and non-villous variants) versus serous carcinoma, (3) endometrioid carcinoma with spindle cells, hyalinization, and heterologous components versus malignant mixed Müllerian tumor, (4) high-grade endometrioid versus serous carcinoma, (5) high-grade endometrioid carcinoma versus dedifferentiated or undifferentiated carcinoma, (6) endometrioid carcinoma with clear cells versus clear cell carcinoma, (7) clear cell versus serous carcinoma, (8) undifferentiated versus neuroendocrine carcinoma, (9) carcinoma of mixed cell types versus carcinoma with ambiguous features or variant morphology, (10) Lynch syndrome-related endometrial carcinomas, and (11) high-grade or undifferentiated carcinoma versus non-epithelial uterine tumors. As carcinomas in the endometrium are not always primary, this becomes the differential diagnosis between endometrial carcinomas and other gynecological, as well as with extra-gynecologic metastases.

Breast cancer patients receiving tamoxifen (Tam) are at an increased risk for developing endometrial carcinomas, possibly due to the partial estrogenic effect of Tam on endometrial cells. Progestational therapy has not routinely been included in Tam regimens. The consistent finding of ER and PR expression in the endometria of post-menopausal women receiving Tam further supports the suspected estrogenic effect exerted by Tam on endometrial cells. Progestational therapy could be beneficial in the prevention of Tam-induced abnormal endometrial proliferations [20]*.*

Mutter et al. showed that 43% of histologically normal pre-menopausal endometria contain rare glands that fail to express the *PTEN* tumor suppressor gene because of mutation and/or deletion. This persists between menstrual cycles. Histopathology of PTEN-null glands is initially unremarkable, but with progression, they form distinctive high-density clusters. These data are consistent with a progression model in which initial mutation is not rate limiting [21].

A physiologic process of *apoptosis* involved in the cyclic growth of normal endometrium [22,23] can be induced by extrinsic factors such as chemotherapeutic drugs and radiation [24]; the oncoprotein Bcl-2 is a well-known regulator of cellular apoptosis, inhibiting physiologic process [25]. Progestin-induced apoptosis may occur during the early period of treatment for endometrial hyperplasia [26]. Fas-Fas binding plays a fundamental role in the regulation of the immune system, triggers apoptosis, and may be involved in the development of endometrial hyperplasia [27]. Bax/Bcl-x may be the major control mechanisms of apoptosis in advanced carcinomas; other members of the Bcl-2 family may also be under hormonal control [28].

Fas-related apoptotic pathway is also involved in the regulation of apoptosis in the endometrial tissue and promotes the development and progression of endometrial neoplasia, considering a significant increase of Fas, caspase-3, and M30 expressions in carcinomas [29].

Transition of endometrial epithelium from hyperplasia to cancer seems to involve both increased apoptosis and decreased Bcl-2 expression. Flow cytometric evaluation of M30 and Bcl-2 expression levels, with SPF, in curettage specimens from post-menopausal patients complaining of bleeding provides a quantitative assessment of endometrial apoptosis, anti-apoptosis, and proliferation. Further studies are needed to determine the relationship among these three processes as indicators of the biological behavior of gynecological tumors [30].

Evidence for the existence of *adult stem*/*progenitor cells* in human and mouse endometrium is now emerging because functional stem cell assays are being applied to uterine cells and tissues [31].

### Polyps

Polyps from the endometrium cause abnormal uterine bleeding, infertility, and pelvic pain [32]. Endometrial polyps undergo cyclic changes in the expression of their proteins related to proliferation and apoptosis during the menstrual cycle, similar to those of the cycling endometrium [33].

The concentrations of ER and PR in the glandular epithelium were significantly higher in endometrial polyp than in the normal endometrium. The concentrations of these receptors in the glandular epithelium and stroma were similar in the post-menopausal and pre-menopausal patients [34].

Mittal et al. concluded that endometrial polyps may be a result of a decrease in ER and PR expression in stromal cells. Because of these receptor-negative stromal cells, endometrial polyps may relatively be insensitive to cyclic hormonal changes [32]; while the concentrations of ER and PR in glandular epithelium were higher in polyps than in the normal endometrium, the concentrations of these receptors in the glandular epithelium and stroma are similar in the post-menopausal and pre-menopausal patients. The study by Peng [35] that measured the expression of hormone receptors (estrogen receptor and progesterone receptor) in endometrial polyps and compared the results to surrounding endometrial tissue in women prior to menopause showed that the expression of estrogen receptor was higher whereas the expression of progesterone receptor was lower than that of the adjacent endometrial tissue. The results suggest that the abnormal expression of hormone receptor contributes to endometrial polyp formation. Fujishita et al. demonstrated the expression of ERs, ER mRNA, and PRs in the columnar cells of the pelvic peritonea and typical endometriosis, but not in the normal mesothelium. These results suggest that endometriosis may originate from the columnar cells with ERs and PRs in the pelvic peritoneal lining [9].

Endometrial polyps can appear in menopausal women receiving hormone replacement therapy despite the presence of progestins to oppose the action of estrogens [36]. Hormone replacement therapy (HRT) impacts on the expression of Ki-67, Bcl-2, and c-erb.B2 in endometrial polyps during menopause and may cause endometrial polyp involution by decreasing proliferation and stimulating apoptosis [37].

*Vereide AB* showed that proteins in the apoptotic cascade are regulated by gestagen; stromal Bcl-2 expression is a potential biomarker which can separate responders of gestagen treatment from non-responders after oral administration [38]. Part of the molecular mechanisms of progestin therapy for endometrial hyperplasia is through the upregulation of Fas/FasL expression [27]. Dysregulation of Fas/FasL expression in hyperplastic endometrium may be part of the molecular mechanisms for non-responders to progestin treatment. Intermittent, rather than continuous, progestin treatment may be more effective clinically for the treatment of endometrial hyperplasia.

Taylor et al. [39] demonstrated three significant differences found between the endometrium and the polyps. Polyps taken from the proliferative phase of the cycle displayed a significantly elevated expression of Bcl-2 and a weak or no expression of progesterone receptors. Secretory phase polyps displayed an elevated expression of estrogen receptors.

A localized increase in Bcl-2 expression and consequential decline or cessation of apoptosis are important mechanisms underlying the pathogenesis of endometrial polyps. Elevated Bcl-2 expression results in failure of the polyp tissue from undergoing normal cyclical apoptosis during the late secretory phase [39-41]. This may mean that the polyp is not shed along with the rest of the endometrium during menstruation.

However, estrogen may have a role in the development of post-menopausal endometrial polyps, either by direct stimulation of localized proliferation or by stimulation of proliferation via other pathways, such as activation of Ki67 or through inhibition of apoptosis via Bcl-2. The c-erbB-2 is unlikely to play any role in the development of these lesions [42]. Ki-67 and c-erbB2 overexpressions are frequent in endometrial polyps in post-menopausal women [43].

Endometrial polyps in menopausal patients receiving HRT respond only to estrogens, but not to progestins. The unopposed estrogenic action on polyps may favor the development of pre-malignant hyperplasia and carcinoma [44].

### Ultrasound

Ultrasound diagnosis has been successfully used to differentiate tumors of the uterus and appendages [45-52]. Transvaginal ultrasound is a cost-minimizing screening tool for perimenopausal and post-menopausal women with vaginal bleeding [45] and is preferred over uniform biopsy of post-menopausal women with vaginal bleeding because it (1) is a less invasive procedure, (2) is generally painless, (3) has no complications, and (4) may be more sensitive for detecting carcinoma than blind biopsy. Transvaginal sonography is rarely non-diagnostic. A limitation of ultrasound is that an abnormal finding is not specific: ultrasound cannot always reliably distinguish between benign proliferation, hyperplasia, polyps, and cancer; that should not be seen as a crucial limitation because tissue sampling is required in either case. Ultrasonography also may be used as a first-line investigation in other populations with abnormal uterine bleeding. In a multicenter, randomized, controlled trial of 400 women with abnormal uterine bleeding by Davidson and Dubinsky [45], the investigators found that transvaginal sonography combined with Pipelle endometrial biopsy and outpatient hysteroscopy was as effective as inpatient hysteroscopy and curettage [45]. Occasionally (in 5% to 10% of cases), a woman's endometrium cannot be identified on ultrasound, and these women also need further evaluation.

Transvaginal ultrasonography has a poor positive predictive value but has a high negative predictive value for detecting serious endometrial diseases in *asymptomatic post-menopausal women*[46]. A limit of M-echo thickness at 8 mm [47] or 10 mm [48] was suggested for this category of patients, as the upper limit for normal thickness was also suggested on a value of 9 mm in women receiving treatments associated with thicker endometria (estrogen alone and cyclical combinations) [49-51].

The negative predictive value for ultrasonography was high (99%) when the threshold for endometrial thickness was 5 mm. This high negative predictive value is not a justification for the use of ultrasonography in screening since 53% of the women with normal biopsies were reported to have an endometrial thickness of at least 5 mm [46].

Nordic multicenter study showed that the risk of finding pathologic endometrium at curettage when the endometrium is <4 mm as measured by transvaginal ultrasonography is 5.5%. Thus, in women with post-menopausal bleeding and an endometrium <4 mm, it would seem justified to refrain from curettage [52]. According to Fleischer et al., the sampling rate of women with an endometrial thickness >6 mm was too low (45%) for confidence interval in the positive predictive value of 2%. Despite a high negative predictive value (99%), transvaginal ultrasonography may not be an effective screening procedure for the detection of endometrial abnormality in untreated post-menopausal women who are without symptoms [53].

Ultrasound imaging of endometrium with atypical hyperplasia in post-menopausal women was found non-homogenous and irregular, and the rarest was in the cases of affirmed fluid in uterine cavity [14].

According to statements of the Consensus of Society of Radiologists [54], the following recommendations were used to create an algorithm for evaluating women with post-menopausal bleeding:

• Because post-menopausal bleeding is the most common presenting symptom of endometrial cancer, when post-menopausal bleeding occurs, clinical evaluation is indicated;

• Either transvaginal sonography or endometrial biopsy could be used safely and effectively as the first diagnostic step. Whether sonography or endometrial biopsy is used initially depends on the physician's assessment of patient risk, the nature of the physician's practice, the availability of high-quality sonography, and patient preference. Similar sensitivities for detecting endometrial carcinoma are reported for transvaginal sonography, when an endometrial *thickness >5 mm* is considered abnormal, and for endometrial biopsy, when ‘sufficient’ tissue is obtained. Currently, with respect to mortality, morbidity, and quality-of-life end points, there are insufficient data to comment as to which approach is more effective [55-57].

A combination of transvaginal sonography, Pipelle endometrial biopsy, and outpatient hysteroscopy (1) has similar efficacy to inpatient hysteroscopy and curettage for the investigation of abnormal uterine bleeding; (2) hysteroscopy will detect some fibroids and polyps missed by a combination of transvaginal ultrasound and Pipelle endometrial sampling; (3) the quality of histological samples obtained by outpatient Pipelle were comparable to those obtained by formal inpatient curettage; and (4) outpatient procedures were well tolerated, with good patient acceptability [58].

### Sonoelastography

Today, a new non-invasive method of examination, *sonoelastography* (SEG), which is based on the ultrasonic examination of tissues softness, is constantly developing. SEG as a tissue strain imaging was first described in 1991 [59]. The phenomenon is based on the fact on inverse scattering ultrasonic signal in mild compression and relaxed (i.e. approximately 2%) insonated tissue during the study. The main advantage of such a diagnosis is its high sensitivity. However, there is still a lack of evidence regarding endometrial assessment using SEG. Thus, Preis et al. [60] in a group of 35 perimenopausal patients obtained a sensitivity value of sonoelastography for endometrium hyperplasia as high as 100%. However, the bigger group of patients has to be analyzed to confirm specificity and accuracy. Recently, we suggested the use of blue-green-red (BGR) sonoelastography artefact as a sign to indicate the presence of fluid content in cavities for predicting the liquid content and the possibility for puncture. Ovarian cyst sonoelastography can be effective for liquid detection and has an 88% positive predictive value [61].

### Treatment

Taking into account the fact that the sensitivity to hormone therapy and prognosis of EH in women is largely determined by receptor status, which depends on the clinical stage and degree of histological differentiation of endometria, the aim of our study was to determine the characteristics of endometrial receptors using immunohistochemical methods [3,8].

On the other hand, we are aware of a percentage of patients whose progestin treatment does not give the desired results; we believe that it is this category of patients that is subject to special individual approach to treatment and observation. After hormonal treatment, EH relapses occurred in 15%, 9% to 27%, and 2% of patients due to the morphological heterogeneity of endometrial proliferation. The sensitivity to therapy and prognosis are largely determined by the receptor status.

Existing methods such as cryosurgery, laser destruction and electrodestruction, and thermoablation may lead to irrevocable destruction of the endometrium. The practice of minimally invasive ablation made possible the removal of the endometrium basal layer.

Hysteroresection of the endometrium is considered to be the most reliable technique for the management of endometrial pathology and uterine bleeding because it provides information on the histologic characteristics of endometrium, removal of the tissue within a prescribed depth, and coagulation of bleeding sites.

Hysteroscopy is likely to become the new gold standard in the future because of its ability to visualize directly the endometrium and perform directed biopsies as indicated. As office-based hysteroscopy becomes more practical and widespread, the technique may become more cost effective. An evaluation plan using transvaginal sonography as the initial screening evaluation followed by endometrial biopsy or, more likely, hysteroscopy is likely to become the standard of care.

In recent years, with the introduction of new endoscopic technology, the range of surgical treatment methods for this category of patients has expanded, particularly for those patients with concomitant somatic pathology. One of these innovative treatments is endometrial ablation, the essence of which is the hysteroscopic removal of the basal layer in order to achieve amenorrhea [3]. Reliable visual control ensures efficiency of minimally invasive technologies in all spheres of gynecologic practices.

It remains unproven whether certain patients at higher risk for carcinoma should proceed directly to invasive evaluation. Patients on tamoxifen with persistent recurrent bleeding, those with significant risk factors for carcinoma, and patients with life-threatening hemorrhage comprise this group. Further studies are still necessary to evaluate high-risk patients and determine whether ultrasound or biopsy is really the most cost-effective initial test [45].

These facts confirm the need to determine a new integrative view assessing the state of receptor systems and sonography data for each case, to reach a *personalized treatment strategy*.

The *aims* of this strategy were as follows:

• to assess the state of receptor systems in endometrial hyperplasia,

• to evaluate the capabilities of ultrasound diagnostics and sonoelastography for diagnosis of endometrial pathology and control minimally invasive treatment,

• to develop algorithm for personalized treatment for patients with endometrial hyperplasia with regard to age, integrative assessment of immunohistochemical, and radiology biomarkers.

## Methods

We included in the study 313 white Ukrainian women aged 25 to 45 years who were treated at the Gynecology Center of the Clinical Hospital ‘Pheophania’ from January 2010 to June 2013; they were divided into the following: group 1 (*n* = 112) with glandular cystic hyperplasia, group 2 (*n* = 98) with endometrial polyps, and group 3 (*n* = 103) with atypical endometrial hyperplasia; 82 women who underwent hysteroscopic investigation for infertility before the cycles of *in vitro* fertilization were included as the control group. Age of women included in the observation group ranged from 20 to 45 years, and the average was 38.0 ± 2.3 years.

Diagnosis of EH was combined with dysfunctional uterine bleeding (82.3%), inflammatory diseases of genitals (77.4%), and endocrine diseases, such as obesity, thyroid disease, and diabetes (35.5%), which, together with sensitivity of maintaining reproductive to hormone therapy functions, were considered for personalized treatment. The patients were distributed into the groups with non-significant difference among groups as regards to age. The *design* of the study was prospective and non-randomized.

All patients underwent general clinical examination, which included clinical and biochemical blood tests; blood tests for HIV, RW, HBS-Ag, and HCV-Ag; clinical urine tests; ECG; ultrasound; chest X-rays; and a study of vaginal biotope (microflora), according to protocols of the Ministry of Health of Ukraine. In the study group, diagnostic search was conducted using ultrasound to maintain hysteroresection. The resulting material was subjected to a histological study to determine the receptor of the endometrium cells applying immunohistochemical method, depending on the outcome of patients who later designed the appropriate medical tactics.

### Ultrasonography

All patients underwent clinical examination, transvaginal ultrasound (US) scanning including sonoelastography and 3D/4D technology. Months (1, 3, 6, and 12) after hysteroresectoscopy, we evaluated the endometrial structure, thickness, margins, with myometrium within the TV US diagnostic protocol. Ultrasound scanning using transvaginal probes of the ultrasound scanner HITACHI 7500 (Tokyo, Japan) with a frequency of 5–8 MHz was carried out before, immediately after, and 1and 6 months after the intervention. To define the sonoelastography patterns and the comparative analysis, we used a visual grading system (grades 1–5), which was adopted according to the color variation. The color scheme was red (soft), green (medium stiffness), and blue (hard). Three-dimensional US imaging was performed on Siemens Elegra (Munich, Germany). The uterus was scanned in the coronal and longitudinal projections. The thickest anteroposterior diameter of the endometrial stripe was measured in the sagittal plane.

### Hysteroresection

Hysteroresectoscopies were performed using an 8-mm unipolar resectoscope (Karl Storz GmbH & Co., KG, Tuttlingen, Germany). After 6 months, we performed a control hysteroscopy with endometrial biopsy to assess therapy effectiveness.

On the first stage, the nature of the pathological process in the endometrium was determined by conducting diagnostic hysteroscopy with mandatory scraping of the walls of the uterus with pathohistological verification of the diagnosis. On the second phase, hysteroscopic endometrial ablation was carried out with subsequent follow-up. During the year, we performed ultrasonography of the endometrium on the control with the definition of the M-echo (at 1, 3, 6, and 12 months after hysteroresectoscopy), which clarified the nature of the menstrual function. Written informed consent was obtained from all patients for the publication of this report and any accompanying images.

### Pathology

Endometrial biopsies were performed mainly in the late proliferative stage phase. Sections were stained with hematoxylin and eosin. Estrogen receptor (SP1), progesterone receptor (SP2), Ki67, and p53 were measured on paraffin sections using the manufacturer's recommended protocol (Thermo Scientific, Waltham, MA, USA), as presented in Table 1. For evaluation of reactions, we used a point scale assessment developed by the manufacturer, and immunohistochemical reaction to receptors counted positive in the presence of at least three points.

**Table 1:** Immunohistochemistry markers

**Marker**	**Clone**	**Catalogue number**
Estrogen receptor (SP1)	SP1	RM-9101-S
Progesterone receptor (SP2)	SP2	RM-9102-S
Ki-67 (SP6)	SP6	RM-9106-S
p53	Y5	RM-2103-S

As a de-masking maneuvre, we heated glasses on a steam bath. For immunohistochemistry reaction, we used rabbit monoclonal antibodies. To visualize the results of immunohistochemical reaction, we used the peroxidase of universal set, UltraVision LP Detection System: HARP Polymer (RUT). Background fabric painting was provided with hematoxylin.

The medical ethics commissions of the Clinical Hospital ‘Pheophania’ of State Affairs Department approved the study. Mann–Whitney U test was used to perform a comparison between groups.

## Results

**Figure 1:**
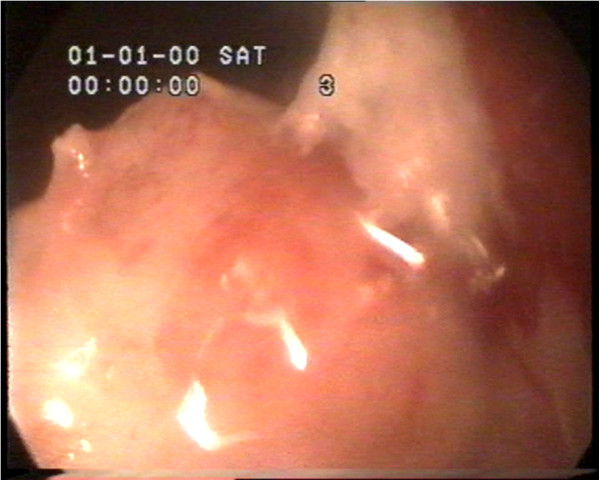
Hysteroscopic image of glandular cystic hyperplasia of the endometrium.

Analysis of the work found that glandular cystic hyperplasia of the endometrium occurs during anovulatory cycles which tend to be longer than the normal menstrual cycle after prolonged persistence of follicles, most of which frequently occur in women 40–45 years who are bleeding prior to amenorrhea for 1 and 2–5 months. The extended phase of anovulatory cycles results from prolonged high concentration of estrogen, resulting in endometrial hyperplasia which is processed as glandular (non-atypical complex hyperplasia) or glandular cystic (simple non-atypical hyperplasia) endometrial hyperplasia. A hysteroscopic image of glandular cystic hyperplasia is shown in Figure 1.

In the histological examination, no distribution on the compact and spongy layers was revealed and glands were unevenly distributed in the stroma; the second type was characterized by cystic expanded glands. Thus, in contrast to *atypical* hyperplasia, the number of glands did not increase, but due to proliferation of the glandular epithelium, each tube was lengthened and had a winding form. Therefore, the histological sections are determined as if the number of glands increased. There are three options for hormonal endometrial proliferation: it could proliferate in the most frequently occurring patterns, characterized by an equal proliferation of glands and stroma (65%); the structure could have a predominance of stromal proliferation (25%); and the structure could be dominated by the proliferation of glands (10%).

**Figure 2:**
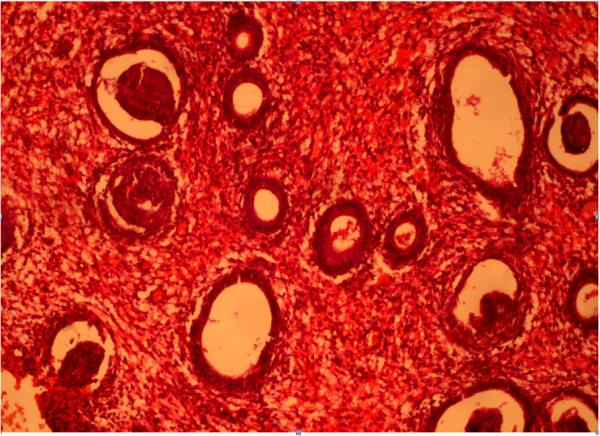
**Glandular cystic hyperplasia.** Coloring by hematoxylin-eosin (×40).

The content of estradiol and progesterone receptors in epithelial cells and stroma with glandular cystic hyperplasia of the endometrium with immunohistochemical study is presented in Figure 2.

**Figure 3:**
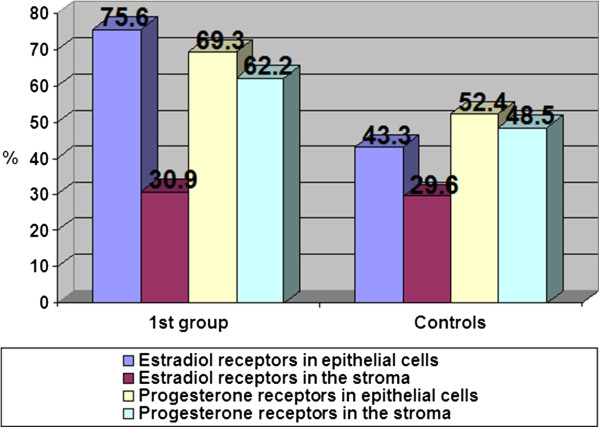
Content of estradiol and progesterone receptors (%) in the endometrium with glandular cystic hyperplasia.

In patients with glandular cystic hyperplasia, the concentration of estradiol receptors in epithelial cells was significantly higher as compared to that in the control group. Thus, the content of estradiol receptors in epithelial cells was 75.6%, while the rate in the control group was 43.3% (Figure 3).

Our findings did not show significant changes in stromal content in these groups (30.9% in the group with glandular hyperplasia and 29.6% in the control group). In our opinion, this is due to the type of hyperplasia. Glandular cystic hyperplasia is usually characterized by irregular proliferation of glandular and stromal components due to irregular activation of the receptor system of glands and stroma; the number of progesterone receptors in epithelial cells and stroma in patients with glandular cystic hyperplasia was slightly higher than in the control group (69.3% in the epithelial cells and 62.2% in the stroma in patients with glandular cystic hyperplasia; 52.4% and 48.5% in the control group, respectively). This fact confirms the role and relative hyperestrogenemia and hypoprogesteronemia at the cellular level for the proliferative state of the endometrium. As the results of our research, the hormone levels do not always determine the degree of proliferation; a significant role in the pathogenesis of endometrial hyperplasia has a certain value of steroid hormone receptors that causes the sensitivity of endometrial cells.

**Figure 4:**
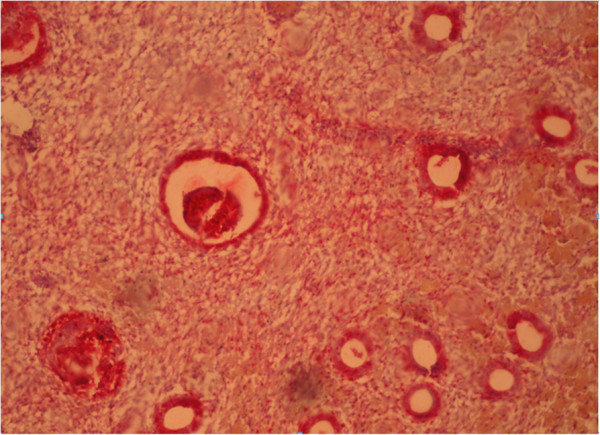
Immunohistochemical reaction in the endometrium with glandular cystic hyperplasia of antibodies with estrogen (×40).

**Figure 5:**
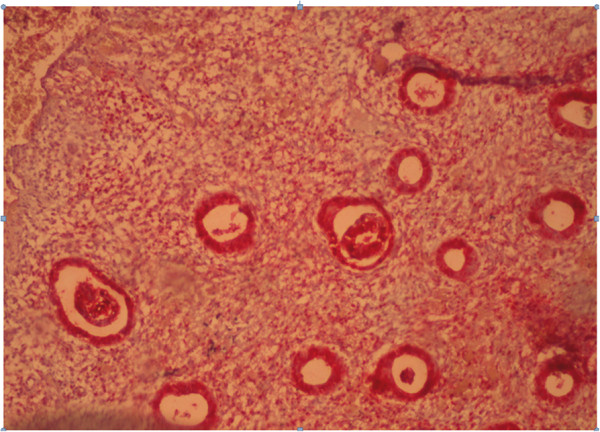
Immunohistochemical reaction in the endometrium with glandular cystic hyperplasia of antibodies with progesterone (×40).

Immunohistochemical reaction in the endometrium with glandular cystic hyperplasia of antibodies with estrogen and progesterone is shown in Figures 4 and 5.

**Figure 6:**
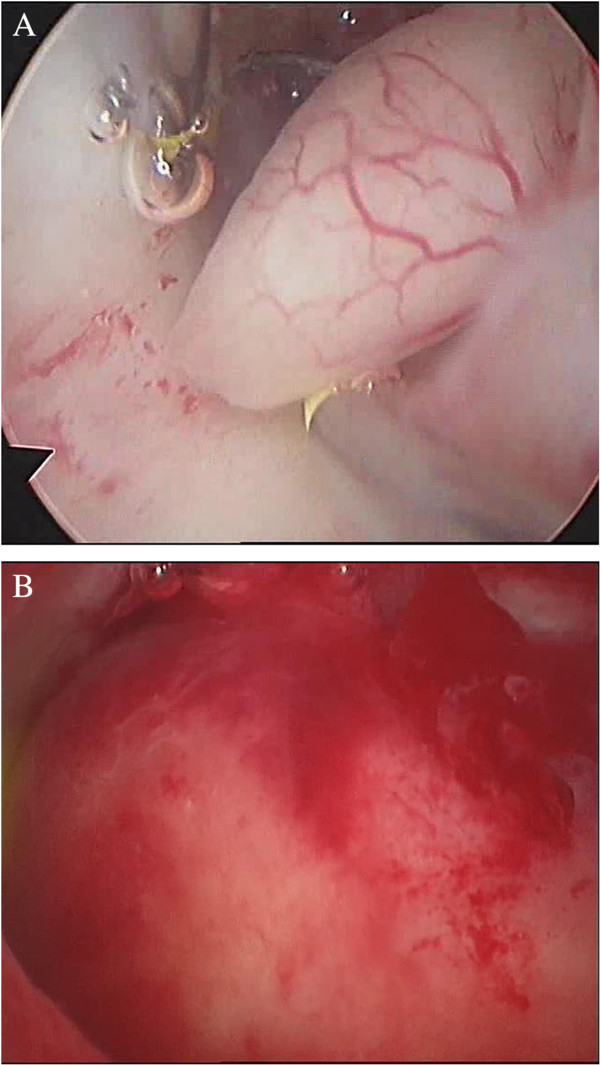
**Hysteroscopy of intrauterine lesions. (A)** Fibrous endometrial polyp and **(B)** submucosal fibromyoma.

While assessing receptor status in the immunohistochemical study, we also determined the level of proliferative activity using the proliferative marker Ki-67. As already was established, glandular cystic hyperplasia in most cases was characterized by increased proliferation of the glandular component and irregular stromal proliferation. This was confirmed when the determining proliferative marker expression was used in glandular cystic hyperplasia. Thus, in functionally active glands, Ki-67 expression increases in some places up to 50% with an almost negative reaction in glandular cystic hyperplasia. Following this concept, we determined the distribution of steroid receptors in patients with endometrial polyps (Figure 6).

**Figure 7:**
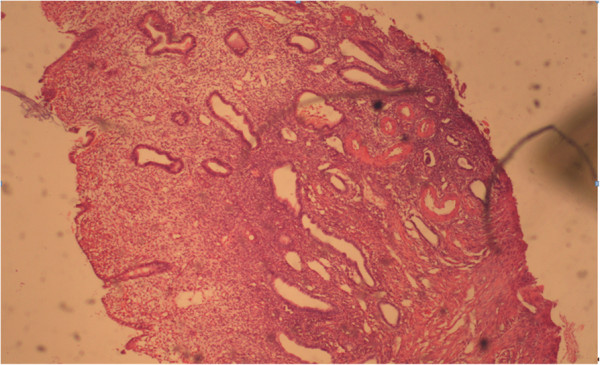
**Glandular cystic endometrial polyp.** Coloring by hematoxylin-eosin (×40).

**Figure 8:**
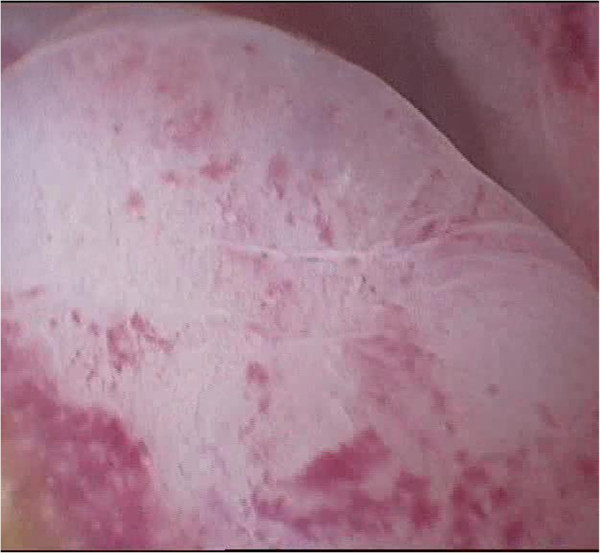
Hysteroscopic image of glandular endometrial polyp.

According to our findings, uterine polyp is a local lesion of exophytic growth, derived from the basal layer of the endometrium. In the early stages of development, polyps look like small proliferates located in the basal section of the endometrium on the verge of the myometrium. Microscopically, these foci are different from the surrounding normal endometrium disordered clusters of tubular and glandular structures with low row indifferent epithelium type, surrounded by a dense cellular stroma. The basal growth area of glandular proliferation penetrates the upper layers of the endometrium, pushing them through expansive growth and bulges above the surface as exophytic lesion. The surface of the polyp is often covered with a layer of functional endometrium that participates in the cyclic changes as the neighbouring endometrium and is rejected in phase desquamation. A hysteroscopic imaged of intrauterine lesions: endometrial polyp and submucosal fibromyoma are shown in Figure 6. Histology of one type of polyp, namely glandular cystic polyp, is shown in Figures 7 and 8.

In analyzing the immunohistochemical data of receptor status in women with endometrial polyps, we found a similar trend in the distribution of receptors in patients with glandular cystic hyperplasia.

**Figure 9:**
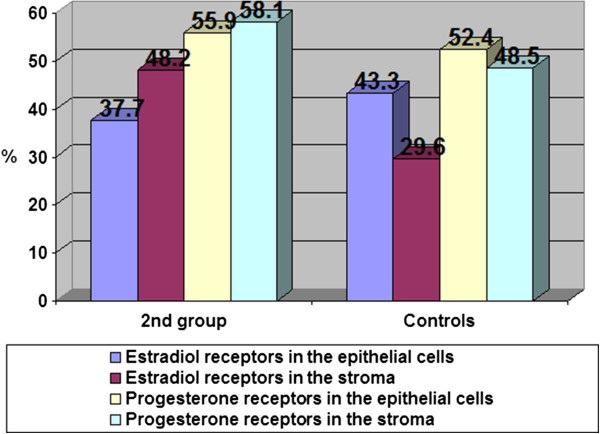
The content of estradiol and progesterone receptors (%) in the endometrium with endometrial polyps.

Thus, in a group of polyps, we have identified some differences: the number of estradiol receptors in the stroma was significantly higher than those in the control group; they were 48.2% and 29.6%, respectively. The content of progesterone receptors in the stroma was 58.1% and 55.9% of endometrial epithelium, whereas in the control group, the content of progesterone receptors in the stroma was 48.5% and 52.4% in endometrial epithelium (Figure 9).

**Figure 10:**
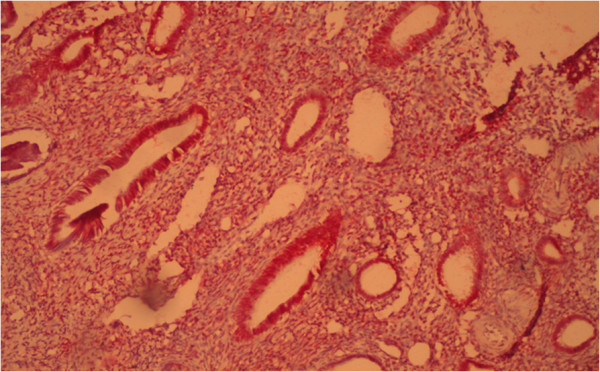
Immunohistochemical reaction in the endometrium with glandular cystic polyp of antibodies with estrogen (×40).

**Figure 11:**
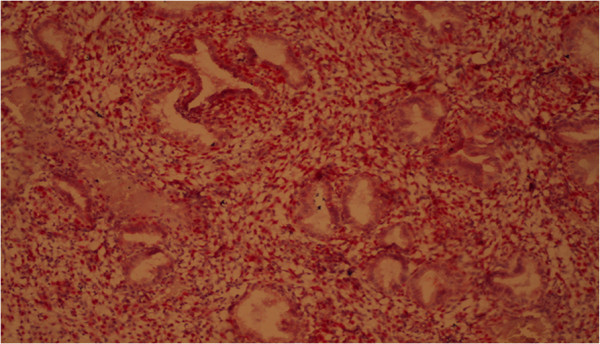
**Immunohistochemical reaction in the endometrium with glandular cystic polyp of antibodies with estrogen (×40)**.

According to conventional pathogenesis paradigms, polyp is a hyperplastic process in the endometrium in response to any stimulation (inflammation, hormonal imbalances, etc.) that is not of tumor origin. The results of our studies demonstrate that an imbalance of receptor status is also relevant for the development of polyps, most notably an imbalance of estrogen receptors in epithelial and endometrial stroma. Immunohistochemical reaction in the endometrium with polyps of antibodies with estrogen and progesterone is shown in Figures 10 and 11.

Pathological proliferation of the endometrium, which loses hormonal hyperplasia characteristics and has the emergence of patterns inherent to malignant tumor, is called *atypical endometrial hyperplasia*. According to the degree of prevalence, diffuse and focal types are distinguished; according to the proliferation of glandular and stromal components, simple and complex forms are distinguished.

**Figure 12:**
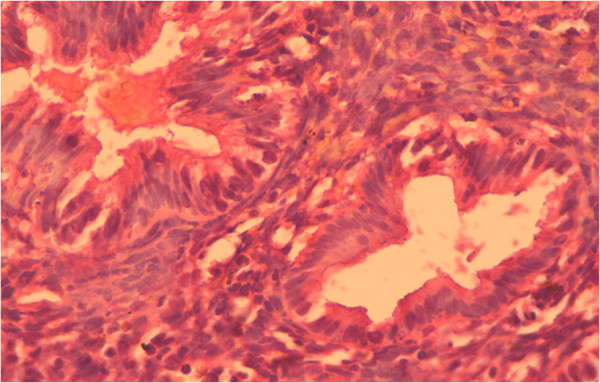
**Atypical glandular hyperplasia of the endometrium.** Coloring by hematoxylin-eosin (×40).

Histologically, we determined in atypical endometrial hyperplasia the glands with numerous ramifications and papillary projections that protruded into the lumen of glands; they are strongly sinuous, with irregular shape. Here and there, glands are closely located to each other without stromal layers and are separated by a narrow strip of connective tissue only. The epithelial cells of glands acquired the features of tumor processes: reduction of nuclear/cytoplasmic ratio, hyperchromatosis, nuclear polymorphism, etc. There were proliferation and increased mitotic activity and abnormal mitosis. Atypical endometrial hyperplasia is shown in Figure 12.

**Figure 13:**
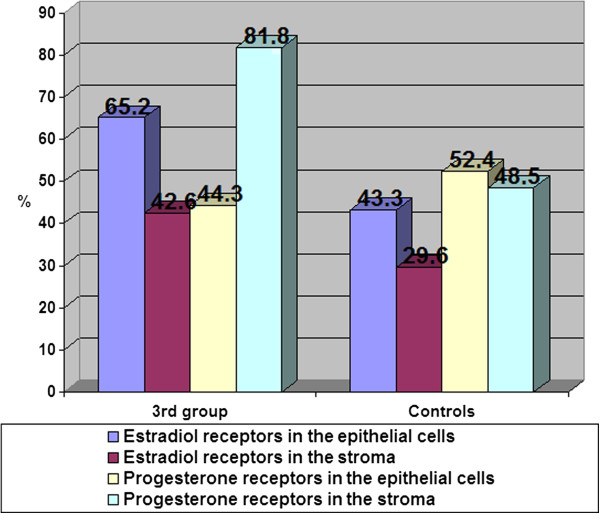
The content of estradiol and progesterone receptors in the endometrium with atypical endometrial hyperplasia.

The content of estradiol receptors in patients with atypical hyperplasia was significantly different from that of the control group, as estradiol receptors in the epithelial cells with atypical hyperplasia amounted to 65.2%; in the stroma, 42.6% (in the control, they were 43.3% and 29.6%, respectively; Figure 13).

There was a significant difference in the content of progesterone receptors in the endometrial stroma (81.8%), which was characterized by a sharp increase in what we believe was a prognostic criterion for determining the subsequent treatment strategy. Percentage of progesterone receptors in epithelial cells was 44.3%, whereas in the control, it was 52.4%. The increased concentration of progesterone receptors in the stroma was probably due to relative hypoprogesteronemia and thus was compensatory anti-proliferative in nature.

However, this trend was not inherent for all observations; in 18.8% patients with atypical hyperplasia, we determined low levels of estrogen receptors on the decreased progesterone receptors' background.

### Ultrasonography data

The study identified the most reliable ultrasound symptoms of EH as follows: heterogeneity of the internal structure; unclear, uneven outer contour; increasing thickness of the M-echo and intensity of endometrial vascularization; fluid in the uterine cavity; polypoid inclusions; and the registration of vascular signals in the subendometrial zone. Ultrasound symptoms of EH are presented in Table 2.

**Table 2:** Ultrasound symptoms of endometrial hyperplasia

**US symptom**	**Glandular cystic hyperplasia**	**Endometrial polyps**	**Atypical endometrial hyperplasia**
Non-homogenous and irregular margins	22%	27%	72%
Mean M-echo thickness	21 ± 2.1 mm	17 ± 1.8 mm	22 ± 2.2 mm
Hypoechoic areas	32%	44%	60%
Fluid in uterine cavity	25%	15%	38%
Hypervascularity on Doppler imaging	15%	42%	57%
RI	0.58	0.7	0.62
Prevalence			
Stiffness (compared to myometrium)	45%	92%	65%
Isoelasticity (compared to myometrium)	35%	8%	27%
Softness (compared to myometrium)	30%	-	7%
BGR	12%	-	45%

**Figure 14:**
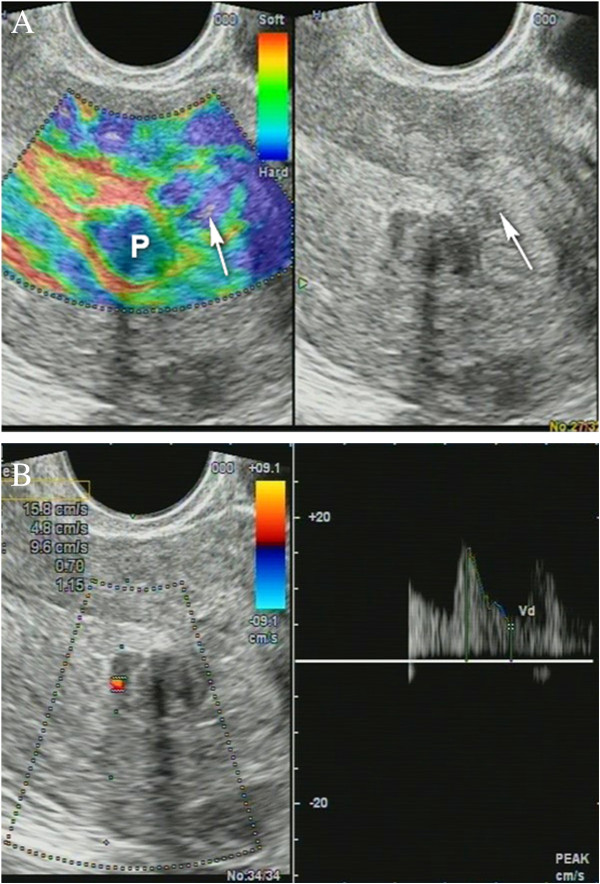
**Endometrial polyp (*P*). (A)** Stiff polyps on SEG, *arrow* indicates invisible polyp in *grey scale* that was detected on SEG; **(B)** Dopplerography of polyp vessel, RI = 0.7.

**Figure 15:**
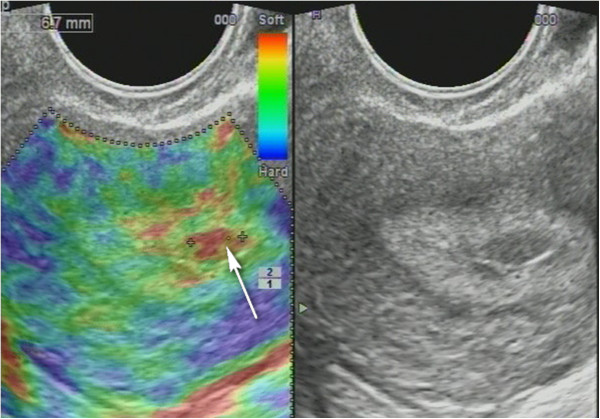
**Sonoelastography of endometrial lesion.***Soft pattern* on SEG helps to exclude the polyp.

**Figure 16:**
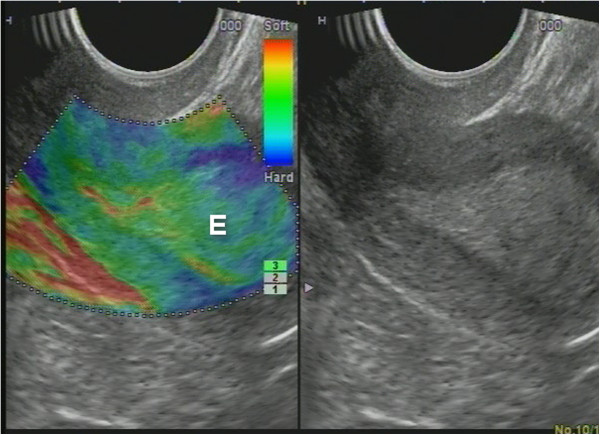
**Glandular cystic hyperplasia that is softer than the myometrium on SEG.***E* endometrium.

**Figure 17:**
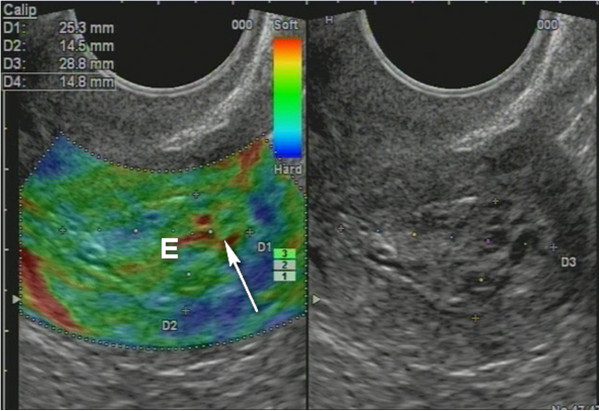
**AEH endometrium (*E*) that is softer than the myometrium on SEG**. Fluid inclusions and BGR artefact (+) are denoted by an *arrow*.

**Figure 18:**
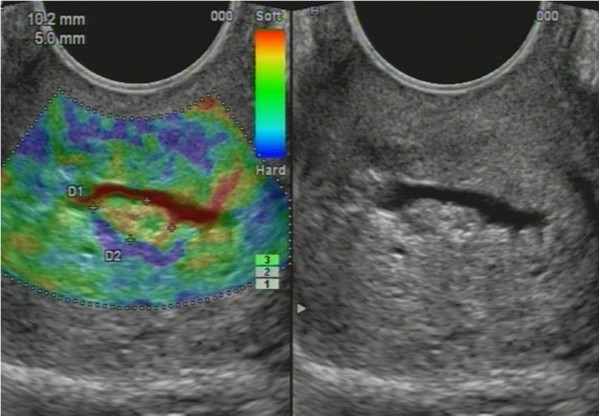
**Polyp fluid in the uterine cavity.** BGR artefact marks a fluid in the uterine cavity in AEH. Sonoelastography appearance of shades of color (*blue*, *green*, and *red*) through stratification artefact - BGR - a sign indicating the presence of fluid in cavities.

Non-homogenous and irregular margins were significantly higher in AEH than in both groups (*P* < 0.01). Mean M-echo thickness was found to be non-significant. Hypoechoic areas, hypervascularity on Doppler imaging, and stiffness (Figures 14 and 15) were more specific for AEH than for glandular cystic hyperplasia (*P* < 0.01, *P* < 0.01, *P* < 0.01, respectively; Figure 16). Fluid in the uterine cavity and BGR artefact were found to be specific for AEH (*P* < 0.01; Figure 17); BGR appearance correlated to the presence of fluid in the uterine cavity (*r* > 0.97; Figure 18).

### Personalization of conservative treatment for AEH

With the main principle in determining the treatment strategy for reproductive age patients which is maintaining reproductive functions as sensitivity to hormone replacement therapy (31% observation), we performed a minimally invasive hysteroresectoscopy loop of 2 mm, with subsequent progestin hormone therapy (endometrin), subsequent ultrasound, and histological control.

In women with endometrial abnormalities, scanty menstruation was relatively rare (up to 3 days, 4.8%); menstrual blood loss is in moderate intensity, lasting 4–6 days (8.0%), is more common, and was found in 21.9% patients.

For patients with low expression of progesterone receptor, we performed a personalized therapeutic strategy considering age and comorbidities. In cases of increased progestin receptors, we administered GnRH agonists (Diferelin) for 6 months; it was followed by recovery of menstrual function and purpose of progestins (4.8% observations).

**Figure 19:**
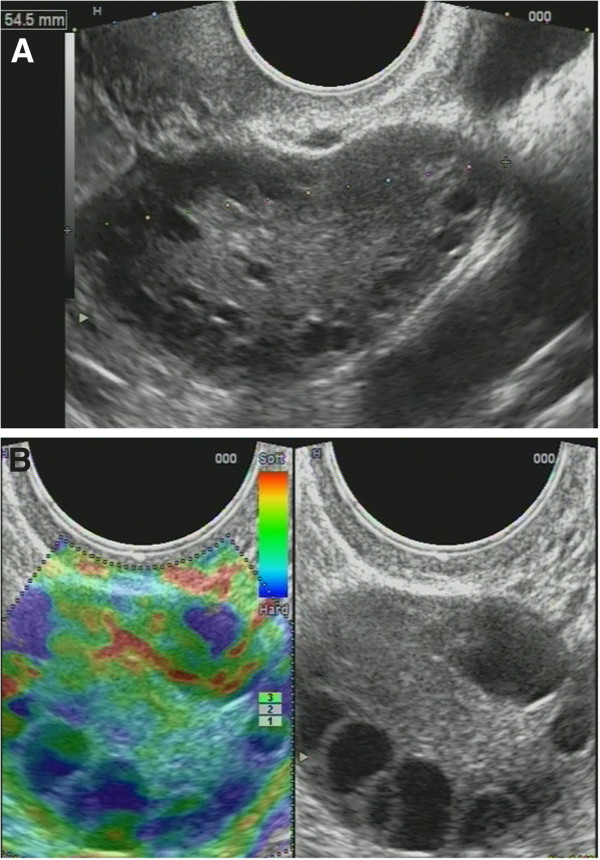
**Ultrasonograms of ovaries. (A)** Polycystic ovarian syndrome (PCOS) - enlarged to 55 mm in length with small fluid inclusions. **(B)** Hyperstimulated ovary: BRG artefact in follicles.

We found that in a group of reproductive-aged women, conservative treatment was effective in 143 patients (45%), and we have observed a normalization of menstrual function and ultrasound characteristics of the endometrium. We considered a dynamic observation of ovary states for conservative therapy (Figure 19).

**Figure 20:**
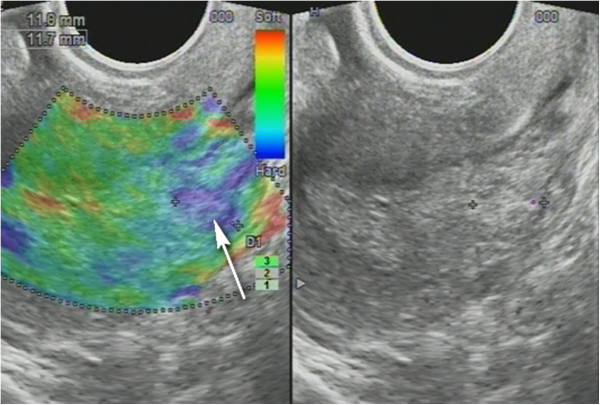
**Polyp relapse.** The personalized image of the guided intervention was considered as the polyp was located close to the thinning of the uterine wall (*arrow*).

In 82 patients (25.8%) older than 35 years, ablative surgery was performed: hysteroresectoscopy to eliminate atypical and basal layers in a single block. In 4.8% of women, we observed EH relapse (polyposis) during progestin therapy, which required re-hysteroresectoscopy, followed by appointment of GnRH agonists (Diferelin) for 6 months and progestins (endometrin). In our opinion, the cause of EH recurrence was insufficient electrodestruction due to the specific anatomy of the uterus (Figure 20). In the 22.6% of patients with AEH and comorbidities (large uterine fibroids, ovarian cystadenoma), hystero/oophorectomies were performed.

### Quality of interventions needs the personalization of minimally invasive treatment of endometrial pathology

Duration of surgery was 34.7 ± 9.3 min on average, while duration of hospitalization was 3.5 ± 1.2 h. The end point was the radical removal of diseased tissue; there was no recurrence of pathological processes in the endometrium. Our research has shown that the use of methods of hysteroscopic endometrial ablation in 88.8% of patients had no endometrial dynamics by ultrasound, reducing the size of the uterus in relation to the original. However, the dynamic follow-up examination at 6 months was found to be 11.2% in women; M-echo increased in thickness, and there was vaginal bleeding from the genital tract, which was the reason for the control of hysteroscopy in order to clarify the state of the cavity cancer and determine the cause of recurrent disease process.

Dissection was done using a hysteroscope and other instruments, and the hysteroscopy control was found to have a uterus filled with adhesions (yellowish white color). Among 38 (11.9%) patients, 28 (8.8%) were detected to have endometrial tissue angles in the uterine tube, and 3.0% of the patients had their endometrium localized in an isthmus area. Regenerated endometrium is marked by single pink islands surrounded by scar tissue. All patients underwent repeat resection of the endometrium. The presence of proliferative endometrial tissue was confirmed morphologically.

In our opinion, the cause of EH relapse was insufficient electrodestruction on specific uterine anatomy. In 22.6% of patients with AEH and comorbidities (large uterine fibroids, ovarian cystadenoma), hystero/oophorectomies were performed.

### Ultrasound for prediction treatment outcome

**Figure 21:**
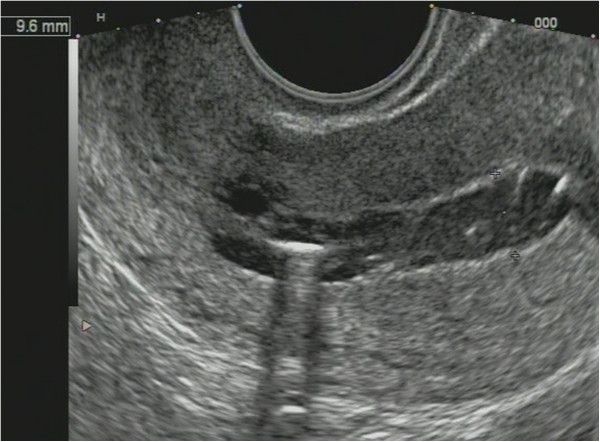
**Uterine cavity after hysteroresection.** Sonogram of unfavorable outcome.

In all patients, hysteroresection was successful with no early complication diagnosed. In 241 patients (75.7%), US showed normalization of the endometrial structure and smooth margins of the myometrium. In 76 patients (23.8%), fibrotic lesions and rough margins of the myometrium were revealed. Endometrial pathology recurrence after 6 month was revealed in seven patients (2.2%) and malignancy in nine patients (2.8%). In 19 patients (5.9%), no US data were found, while clinical symptoms (e.g., uterine bleeding) called for intervention. False negative US results were noted in six patients (2.8%) (Figure 21).

### Traumatic injuries of the myometrium

**Figure 22:**
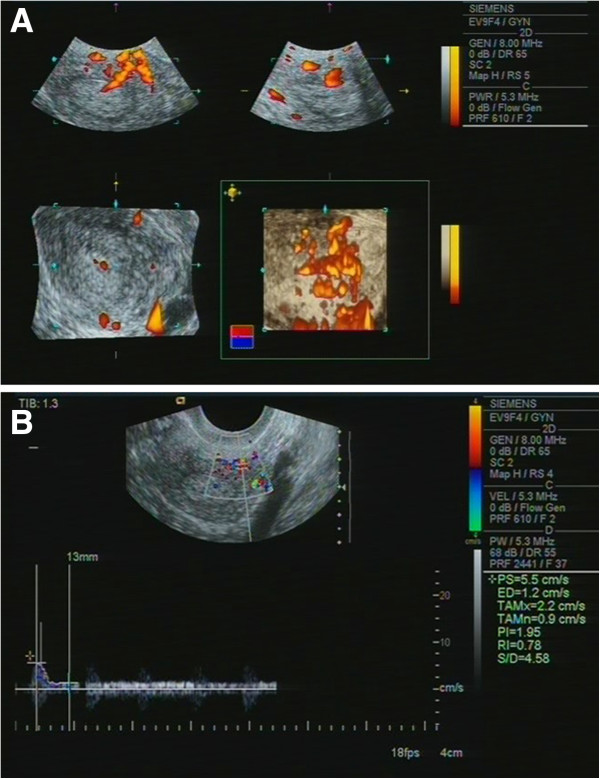
**Arteriovenous malformation in the anterior myometrium wall.** Hysteroscopy was performed 7 months ago. **(A)** In a three-dimensional ultrasound angiography on the anterior myometrium wall, the tortuous vascular plexus is determined with interconnected arteries and veins. **(B)** The blood outflow from the plexus artery to the vein in the endometrium with spectral arterial flow (speed up to 4 cm/s) changed by venous flow (up to 1 cm/s) while transducer was kept in fixed position.

In two patients, after 6 months, *arteriovenous malformation* (AVM) was revealed. AVM can occur after uterine curettage or surgery. After a traumatic injury, pseudo-aneurisms can occur as acquired arteriovenous malformation, arteriovenous fistula, and direct rupture of blood vessels (Figure 22).

### Three-dimensional model-guided approach for the optimization of patient-specific operating technique

The causes of disease process recurrence, according to our study, were a number of factors, namely the lack of resection and electrodestruction in corners of the uterus, due to anatomical features (deep corners, the presence fibromatous nodes, distorting the angle region of the uterus), and the lack of degradation of the mouths of the fallopian tube ball electrode and the neck-loop-peresheechnogo uterine segment electrode. The analysis of this work has allowed us to identify the risk of recurrence of EH on the survey stage and, in the future, to make adjustments to the operational technique of hysteroscopic endometrial ablation.

According to expert recommendations from World EPMA Congress 2011 in Bonn, it recommended to implement the concept of model-guided medicine (CARS [1]).

We apply a three-dimensional modeling based on ultrasound data segmentation and conjoin the models [62] with those created from different source data of visual information (CT, MRI, and post-operative photogrammetry) in a single three-dimensional environment for planning intervention under the ultrasound guidance in real time. The three-dimensional modeling becomes a base of initiation for the model-guided interventions on female genitals [62].

### Sonosurgery

Sonosurgery [63] is a collection of minimally invasive surgical techniques performed with continuous ultrasound imaging and the use of endoscopic tools. It is a surgical discipline which requires the compliance of aseptic and medical art conditions and should be performed in the operating unit by experienced personnel. By medical art, we understand mastery in surgical techniques and ability to perform ultrasound examination by a physician. However, the simplicity and minimal tissue trauma in sonosurgical procedures will lead them to be done in an operating room, similar to procedures in interventional ultrasonography. Sonosurgical techniques are performed just like conventional surgery and orthopedics, but the use of ultrasound equipment can reduce the operating duration and reduce invasive procedures to affected tissues.

## Discussion

Considering anti-proliferative properties of progesterone in relation to endometrium, such levels should probably be regarded as a phase of exhaustion of compensatory processes, which we believe is the subclinical stage of transformation of atypical hyperplasia to the endometrial carcinoma. A slight decrease in estrogen receptor in the transformation of carcinoma can be explained by the disappearance of the biological need for the external support of proliferative activity in case of malignancy due to damage in the genetic apparatus and run their own system of uncontrolled cell growth in the endometrium. These data confirmed the results of the determination of the proliferative activity of the proliferative marker Ki-67; it was determined to have a significant increase of 40% to 50% in the epithelium of the glands while no changes in the stroma were revealed, as compared to glandular hyperplasia.

Thus, our analysis of the ratio of receptor in tissue and endometrial stroma and observation of patients during personalized treatment allowed us to conclude as follows:

1. In glandular EH, the concentration of estradiol receptors in epithelial cells was 75.6% and 30.9% in the stroma, whereas the rate in the control group was 43.3% and 29.6%, respectively, indicating the sharp increase (1.8 times) of estrogen receptors in the endometrium. Analysis of the distribution of receptors for progesterone showed them having a slight increase in the endometrium and in the stroma (1.3 times).

2. Distribution of receptor systems in endometrial polyps characterized by a significant increase in the number of estradiol receptors in the stroma which was significantly higher than that in the control group was 48.2% and 29.6%, respectively (1.7 times). The content of progesterone receptors in the stroma was 58.1% and was 55.9% in the endometrial epithelium in the control group; the content of progesterone receptors in the stroma was 48.5% and was 52.4% in the endometrial epithelium.

3. Receptor-negative feature of patients with atypical endometrial hyperplasia has a significant difference in the content of progesterone receptors in the endometrial stroma (81.8%). The content of estradiol receptors in patients with atypical hyperplasia was significantly different from that of the control group, namely estradiol receptors in epithelial cells with atypical hyperplasia amounted to 65.2%; in the stroma, 42.6% (in the control, 43.3% and 29.6% respectively). This receptor combination, we believe, is the prognostic criteria for determining the subsequent treatment strategy as the method of screening for uterine cancer pathology.

4. On the basis of immunohistochemical studies, with a certain level of receptors for estrogen, progesterone, and proliferative marker Ki-67, it is an undoubted fact that the carcinogenesis of endometrial tumors plays an important role not only on the hormonal status (hormone levels in the blood), but also on the so-called receptor imbalance directly in the endometrium. From the morphological point of view of the sharp variations in the receptor status of the endometrium, it can be interpreted as a risk factor for the development of mutations in the genetic apparatus and thus tumor development.

5. In relation to further management of patients with different types of receptor status, we believe that increasing the percentage of patients having a decrease in both receptors for progesterone and estrogen causes endometrial hyperplasia. In fact, inefficient use of hormone therapy in these patients underscores the need not only for histological and hormonal studies but also for complements of the diagnostic algorithm for determining relationships in the endometrial receptor system that will allow pathogenetic therapy. Thus, each individual pathological pattern of the definition of receptors and their relationship further defines personalized pathogenetic tactics, tailored to the person.

6. Ultrasound has sensitivity, specificity, positive and negative predictive values, and accuracy which were 96%, 85%, 82%, 94%, and 84%, respectively, for early detection of endometrial hyperplasia and prediction outcome of a minimally invasive treatment program. SEG had a sensitivity and specificity of 91% and 83%, respectively, for polyp diagnosis.

7. The study of hormone receptor status in patients with EH allows to clearly define the treatment policy and to avoid relapse, optimizing treatment and observation of such patients.

8. Pharmacotherapy combined with minimally invasive surgery enabled to treat patients with EH and significantly reduced radical interventions and time of treatment.

9. Performing conservative interventions and personalized pharmacotherapy with subsequent use of GnRH agonists and progestin on the second stage is an effective treatment of recurrent EH, which enabled significantly to reduce the number of radical interventions and time of treatment.

### Consolidation of the PPPM concept

Thus, our analysis of the ratio of receptors in the tissue and endometrial stroma in observed patients allowed us to conclude as follows.

#### Personalized medical approach

Each individual pathological pattern of the definition of receptors and their relationship combined with US biomarkers defines further *personalized* tactics. Three-dimensional model-guided approach is necessary to perform case-specific intervention.

#### Predictive medical approach

These receptor combination patterns are *predictive criteria* for determining the subsequent treatment strategy as the method of screening for uterine cancer pathology. The sharp variations in the endometrium receptor status can be interpreted as a risk factor for development of genetic mutations and carcinogenesis.

#### Preventive medical approach

Our results may lead to the initiation of programs to prevent endometrial cancer and improve the quality of life. It is recommended to promote programs for the introduction of ambulatory ‘office’ of hysteroscopic operations that will raise the operative outpatient gynecological care to a new level of efficiency and safety.

### Study limitation

Quantitative shear-wave sonoelastography and contrast-enhanced US were not applied in this study. Particularly, genetic and cellular mechanisms were out of assessment. The study was non-randomized and non-blinded. The confounders as collateral pathologies were present and not profoundly studied.

### Future outlooks and expert recommendations

We suggest further studies to include virus status, immune response, gene damage, and actions of promising substances as nanomaterials (e.g., nanoceria, nanogold) for complex impact on *female health* and collateral pathologies, and to initiate comparative studies to establish science-based treatment algorithms and updated screening programs. After approval, they should develop safe and effective personalized treatments.

### Collateral pathologies related to the studies

Metabolic disturbances in obesity causes a number of diseases, namely cardiovascular diseases, and a number of tumor sites of lung cancer, breast cancer, uterine cancer, and ovarian cancer; in women, there is a violation of ovarian menstrual cycle called dyslipidemia. Obesity reduces life expectancy by 3–5 years, and sometimes, in severe forms, for 15 years [1,2]. The incidence of endometrial cancer is related to increasing age and, with 39% of cases, is attributed to obesity [64]. The recent data, regarding women's differential responses to lifestyle changes, support another branch of research with gender nutrition emphasis within predictive, preventive, and personalized medicine [65].

Illustrated were extensive interrelations among viral action, cellular oxidative stress, gene damage, multiple immune pathways and proteomic changes in cancer related to diabetes mellitus, and many chronic disorder developments [66]. Many of the chronic diseases are also related to virus infection (human papillomavirus (HPV), herpesvirus), followed by gene damage and immune mechanisms involvement [66,67]. HPV infection is attributed to 80% of all human cancers and was supposed to play a central role in the development of breast cancer [66,68]. It was described as a novel improved multimodal diagnostic approach for breast cancer risk assessment, which utilizes a combination of conventional, analytical methodologies for the creation of pathology-specific biomarker patterns [2,68]. Many endocrine, neurologic, autoimmunity, osteoporotic, and neurodegenerative diseases [69] are strongly related to the hormonal status in women.

### Expanding the immunologic study

The study is promising to signal pathways imbalance of pro- anti-inflammatory cytokines, toll-like receptors in carcinogenesis, and cancer relapse in virus-induced malignancies. Considering the ability of group factors, e.g., probiotics, which can promote effective immune response and initiate an effective immune defence, probiotic/immunobiotic application might be promising while integrating the personalized approach.

### Biotherapy

The study of different molecular pathways and the correlation of immunohistochemical findings with histologic grade and clinical stage could help in predicting biologic behavior and planning treatment in patients who are diagnosed as having these tumors [16]. The fundamental studies on endometrial stem/progenitor cells may provide new insights into the pathophysiology of various gynecological disorders associated with abnormal endometrial proliferation, including endometrial cancer, endometrial hyperplasia, endometriosis, and adenomyosis [31].

### Genetic studies

Discovery of somatically mutated cells in human tissues has been less frequent than would be predicted by *in vitro* mutational rates [21,67].

### Nanotechnologies

Nanoparticles of cerium dioxide and gold [70,71] were reported against oxidative damage, working as anti-ageing agent. Treatment with nanoceria results in the increase in the number of oocytes in follicles at metaphases I and II, increase in the number of living granulosa cells, and decrease in the number of necrotic and apoptotic cells [70]. In combination with anti-cancer theranostic application, it is a promising direction to develop in PPP gynecology and reproductive medicine.

### Mathematical modeling approach

Most processes found in medicine are non-linear, chaotic, and have a high level of complexity; creating a reliable mathematical model and use of information technology at all stages of the treatment process from the expression of the pathological processes to the implementation of therapeutic interventions associated with patient and physician perception of these phenomena, and making decisions in the absence of input parameters for the creation of self-controlled systems based on forecasts of future medical errors are important tasks [72]. Previously, we reported [73] the approach solving combinatorial (correctable) problems of selection options of negative prognostic indicators for interventional radiology/surgery mistakes to ensure a high level of patient safety as well as study-level skills and minimal training required for training programs for interventional medicine by applying the *stochastic method of branches and boundaries*. We suggest that this study would have a follow-up to assess the multi-parameter data by novel mathematical model according to which the medical process is recognized as a complex system like the ‘black box’ [74]. This process (EH progression) is described by some of the primary indicators (US and immunohistochemical biomarkers). Thus, primary indicators and output rate are stochastic in nature and are presented as statistical information. The ‘best’ mathematical model of the medical process is studied using a special algorithm for processing statistical data [2]. We would recommend the application of one minimal model to solve tasks as simple as possible.

*Fractal geometry* is a promising opportunity, especially the application of fractal analysis in complex systems of visual diagnostics including radiology and histology data in order to expand its diagnostic capabilities by increasing the information content for intelligent decision modeling to reduce subjectivity in the perception and interpretation [75]. This approach was successfully applied for hepatic oncology [76].

### Education for preventive measures

Educational programs and individual preventive facilities are the important task for the PPPM concept in women's health. Dissemination of information is necessary in order to popularize screening programs and patient participation approaches against risk factors including obesity, fat diet, diabetes, menstrual cycle disorders, alertness in never having been pregnant, receiving hormonal therapy, endocrine pathology, cancer prehistory, and family history. The material for dissemination and lecturing should be standardized (well translated, to be easily understood) in order to facilitate the work. Support of preventive educational activity with long-term commitment of private and public funding programs is required.

### Potential economical impacts

The cost of new gynecological cancers in developing countries in 2009 totalled to US$1.087 billion compared to the US$11.913 billion spent in developed countries [77]; in 2009, cancer costs in the European Union (EU) were estimated at €126 billion, in particular, for corpus uteri, it was €4.554 billion. The preventive campaigns for organizational diagnostic tests/programs with focus on prediction and prevention are available at low costs (ultrasound, most valid biomarkers) and should valuably benefit the economy.

Obesity as a condition associated to endometrial pathology and uterine cancer accounts for the burden related to the treatment of these preventable diseases (about €59 billion a year in EU; US$71.1 billion in the USA). The combined medical costs attributable to obesity and overweight are projected to double every decade and will account for 16%–18% of the total US healthcare expenditure by 2030 [78]. Thus, considering integrative medical approach within the PPPM paradigm directed to women's health should lead to significant *indirect economic benefits*.

Promoting programs for the introduction of personalized outpatient gynecological care as the patient-centerd medical home (PCMH) model based on prediction and prevention is expected to be more cost effective than treatment on advanced diseases in large centers.

With the concluding points, we can formulate the following proposals (expert recommendations):

1. For the EU, an international women's health project including the study of integrative diagnosis and treatment of endometrial pathology in regards to preserving the reproductive function should be created. There should be a sufficient evidence study to determine relationships in endometrial receptor system, genetics, immune pathways to complement the diagnostic algorithm that will allow the development of novel treatments and model-guided approach.

2. For Ukraine, it is recommended to promote programs for the introduction of personalized outpatient (office) gynecological care as the patient-centered medical home (PCMH) model for healthcare delivery with a high level of efficiency and patient safety; it is also recommended that there should be project participation in partnership with the EU to follow up experimental and clinical trials and to involve related institutions and centers to the study.

## Conclusions

The study of hormone receptor status combining with US/sonoelastography data in patients with endometrial hyperplasia allows for the clear definition of the treatment policy and reduction of relapse. Transvaginal sonography in complex application with sonoelastography is a highly diagnostic screening test for endometrial pathology. Performing hysteroresectoscopy and the subsequent use of GnRH agonists (Diferelin) and a progestin (endometrin) in the second stage is an effective treatment of recurrent EH and significantly reduces radical interventions and time of treatment. Pre-operative ultrasound/radiology data analysis allows the following: to simulate the upcoming surgery, to set the anatomical features of the pathologic process, to prevent intra-operative complications, and to identify areas of risk for the development of recurrence.

## Abbreviations

AEH: Atypical endometrial hyperplasia; AR: Androgen receptor; EH: Endometrial hyperplasia; ER: Estrogen receptor; PCOS: Polycystic ovarian syndrome; PPPM: Predictive preventive, and personalized medicine; PR: Progesterone receptor; UPSA: Uterine papillary serous adenocarcinomas.

## Competing interests

The authors declare to have no competing interests.

## Authors’ contributions

VMG was responsible for the idea of the study and the study organization; performed diagnosis, treatment of patients, and data analysis; and prepared the article. VAB participated in the study organization and analysis of the study. OVK performed the immunohistochemical survey. OMD participated in the examination of patients and in data analysis. MYS did the analysis of the study and participated in preparing article. RVB participated in the study organization, in the diagnosis, and in the treatment of patients; performed ultrasound survey; performed literature review; participated in the analysis of the study; described interdisciplinary systematization and prospects; and drafted the article. All authors read and approve the manuscript.

## Authors’ information

VMG is a doctor of medicine and philosophy and is a medical doctor in Center of Gynecology in the Clinical Hospital ‘Pheophania’ of the State Affairs Department. VAB is a doctor of medicine, philosophy, and science and is a professor at the Third Department of Obstetrics and Gynecology in Bogomolets National Medical University, Kyiv. OVK is a doctor of medicine and philosophy and is the head of the Department of Pathology in the Clinical Hospital ‘Pheophania’ of the State Affairs Department. OMD is a doctor of medicine and philosophy and is medical doctor at the Kyiv Perinatal Center and JSC SPC ‘DiaprofMed’. MYS is a doctor of medicine, philosophy, and science; is a professor and corresponding member of the National Academy of Sciences of Ukraine; and is the director of the Inteferon Department of Zabolotny Institute of Microbiology and Virology, NAS of Ukraine, Kyiv. RVB is a doctor of medicine and philosophy; is a medical doctor in the Center of Ultrasound diagnostics in the Clinical Hospital ‘Pheophania’ of the State Affairs Department; and is the National Representative of the European Association for Predictive, Preventive and Personalised Medicine (EPMA) in Ukraine.
